# Thickness measurement of transparent liquid films with Paraxial Self-Reference Interferometry

**DOI:** 10.1038/s41598-020-65799-z

**Published:** 2020-06-08

**Authors:** Ahmad Razzaghi, Jafar Mostafavi Amjad, Maniya Maleki

**Affiliations:** 1 Physics Department, Institute of Advanced Studies in Basic Sciences (IASBS), Zanjan, 45137-66731 Iran; 2 Optics Research Center, Institute of Advanced Studies in Basic Sciences (IASBS), Zanjan, 45137-66731 Iran

**Keywords:** Optics and photonics, Physics

## Abstract

In this paper, we introduce a non-invasive optical method, named Paraxial Self-Reference Interferometry (PSRI) for thickness measurement of liquid films. The method can be used for thin or thick layers (from μm to mm) of solids or liquids, with a high precision. The method is first applied to solid plates with known thickness and is verified to be accurate. Then we use it for the thickness measurement of liquid films in two experiments. The first experiment is spin coating and the second is dip coating. In both experiments, the results are in agreement with theoretical and experimental results of previous works. In the dip coating experiment, the Landau-Levich-Derjaguin law (LLD) is observed in low capillary numbers, and a deviation from this law due to gravity is seen in higher capillary numbers. The thinning due to the drainage is also observed and is consistent with theoretical predictions.

## Introduction

Liquid films formed on solid surfaces are very common in nature and everyday life and have many technological and industrial applications. They are present in processes like coating, lubrication, painting and printing. Controlling and measurement of the thickness of liquid films is essential in all the applications. For transparent films, many optical methods are used for thickness measurements, such as phase shifting interferometry and equal-path interferometer^1,2^, astigmatic method^3^, multiple beam interferometry^4^, dispersive white-light spectral interferometry^5^, moire technique^6^, spectroscopy^7^, and confocal microscopy^8,9^.

The advantage of optical measurement methods is that they are usually non-invasive and precise. The thickness range is from nanometer to micrometer in most of the interferometry methods. For films thicker than micron, confocal microscopy can be used for thicknesses up to a few hundred microns. Unlike the solid films, thickness measurement of liquid films in the range of micron to millimeter is not convenient, because the methods which are non-invasive for solid films, can be invasive for liquid films. Besides, in most of the liquid film experiments, the dynamics of the film necessitates a method with a high time resolution.

In this paper, we introduce a non-invasive optical method, named Paraxial Self-Reference Interferometry (PSRI) for thickness measurement of liquid transparent films. The proposed PSRI method is a powerful technique in thickness measurement of thin films with a high time resolution which makes it useful for dynamic experiments. This robust and fast thickness measurement method is based on single shot data analysis by using of fringe-tracing technique. Unlike to phase-shifting and Fourier-transform techniques, in PSRI method just a single interference data required to generate online thickness measurements. Also, because of its simplicity and low number of optical components that used in PSRI optical setup, the sensitivity of experimental data to the mechanical vibrations, temperature fluctuation and the environmental electrical noise is minimized and the accuracy of experimental reproducibility is increased. Unlike techniques like moire or many other spectroscopy or interferometry methods, which can only measure the thickness difference and need a reference thickness to give the absolute value of thickness, our method measures the absolute value without needing any reference point. The time resolution can be simply adjusted by the frame rate of the camera, and does not require any dynamic part in the setup like the spinning disk in the confocal microscopy. PSIR can be used for thickness measurement of solid and liquid transparent films in the range of micron to millimeter, where other methods are usually not applicable. To check the precision of the method, we first apply it to solid glass plates with known thickness, and observe that the measurements are in a very good agreement with the known thickness. Then we use it for liquid films.

Two techniques have been used for making liquid films in our experiments. The first method is spin coating. The spin coating is a method used to deposit uniform thin liquid films on a flat horizontal substrates. A drop of the liquid is deposited on the substrate and then substrate is rotated with a constant angular velocity. The final thickness of the film is determined by the rotation speed and the viscosity of the liquid^10–16^. We used silicon oils with three different viscosities and measured the thickness of the resulting films (10–170 *μ*m) as a function of angular velocity. The results were in agreement with theory. The second method that we used was a dip coating. Dip coating consists of dipping the substrate inside a liquid reservoir and pulling it out of the bath. The resulting film has a thickness determined by the viscosity and the surface tension of the coating liquid, as well as the pulling velocity. We measured the thickness of films resulting from dip coating in different velocities and for different viscosities, in the range of 10–250 *μ*m. The measurements were consistent with theoretical and numerical predictions.

The PSRI method for thickness measurement enables us to measure thicknesses in a very wide range of 10 *μ*m to $3$ mm and with a good precision of about 3 *μ*m. The advantages of this method include the fast and robust measurement with good accuracy. In the presented method, the optical setup is very simple and accurate by use of Paraxial Self-Reference Interferometry (PSRI) method, and the errors resulting from the physical vibrations have been minimized. The proposed thickness measurement technique, which works based on geometrical and interferometry methods is a powerful method for increasing the measurement accuracy and it can be used in many areas of research.

## Theory of liquid film thickness

There are two main methods for making uniform liquid films on solid substrates: spin coating and dip coating. In spin coating method, a drop of liquid is placed on a horizontal spinning disk and gradually forms a thin film. Balancing the forces in the film, namely the centrifugal and viscous forces, one can obtain the film thickness $t$ as a function of time $\tau$, spin velocity $\omega$ and liquid characteristics^10–16^

1 $$t=\frac{{t}_{0}}{{\left(1+\frac{4{\omega }^{2}{t}_{0}^{2}\tau }{3\nu }\right)}^{1/2}},$$

where ${t}_{0}$ is the initial film thickness, and $\nu$ is the kinematic viscosity of the liquid. This equation shows that in large times, ignoring the 1 in the denominator, we have $t\propto {\omega }^{-1}$.

In dip coating technique, a solid plate is withdrawn from a liquid reservoir with a constant velocity $V$. The thickness of the film formed on the solid is usually expressed in terms of the capillary number $Ca$, which is defined as

2 $$Ca=\frac{\eta V}{\gamma },$$

where $\eta$ is the viscosity and $\gamma$ the surface tension of the liquid. Landau, Levich and Derjaguin calculated the thickness of the film as a function of $Ca$, for $Ca\, <  < 1$. They found that in this regime, gravity can be neglected and the thickness $t$ can be written as^17,18^

3 $$t=0.94\,{\kappa }^{-1}C{a}^{2/3},$$

where ${\kappa }^{-1}$ is the capillary length defined as

4 $${\kappa }^{-1}=\sqrt{\frac{\gamma }{\rho g}}.$$

This law is usually referred as LLD law. When $Ca$ approaches unity, the LLD law is not valid anymore, because it neglects the gravitational force that tends to thin the film. The theories suggest that the thickness deviates from LLD for $Ca > {10}^{-3}$ and predict a lower thickness than LLD in this regime^19–22^. The most precise work was done by Jin *et al*.^22^, in which the complete two-dimensional Navier-Stokes equation has been solved numerically.

Due to gravitational entrainment, the vertical film formed in dip coating thins from the top as time progresses. The thickness profile of the film is given by the Reynolds thinning law^23^

5 $$e(z,\tau )=\sqrt{\frac{\eta z}{\rho g\tau }},$$

where $\tau$ is time, $z$ is the vertical distance from the top of the film, and $g$ is acceleration due to gravity. This profile attaches to the constant-thickness film, at the location

6 $$L(\tau )\approx \rho g{t}_{0}^{2}\tau /\eta ,$$

where ${t}_{0}$ if the initial thickness of the film^24^.

## Optical analysis of PSRI

**Figure 1:**
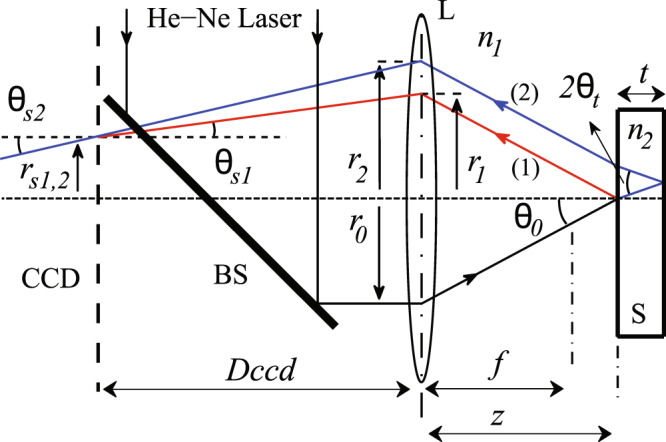
Experimental setup of PSRI.

The propagation behavior of plane waves in a Paraxial Self-Reference Interferometer (PSRI) can be analyzed using ray-tracing method. As shown in Fig. 1, in a PSRI, the collimated beam from a He-Ne laser passes through a beam splitter (BS) and an interferometer lens (L), and then is reflected from two sides of a sample S. Thus, there are two rays that will meet at the same point on the detector plate (e.g. ${r}_{s1,2}$ on CCD). Here, $f$ is the focal length of the interferometer lens L; $z$ and $Dccd$ the sample and detector distance from the lens L; ${n}_{1}$, ${n}_{2}$ refractive indices of air and the specimen; $t$ sample thickness; and ${r}_{0}$, ${r}_{\mathrm{1,2}}$, and ${r}_{s\mathrm{1,2}}$ the distance of the incident, reflected, and interfered rays from the optical axis, respectively.

### Ray tracing

We used paraxial ray-tracing matrix method to compute the phase difference between two interfering rays. In this method, the intersection position of two interfering beams on the detector plate is calculated. We assume that the incident beam with position (${r}_{in}$, ${\theta }_{in}$) passes through PSRI setup and the interfering rays positions (${r}_{s\mathrm{1,2}}$, ${\theta }_{s\mathrm{1,2}}$) will be derived as

7 $$\begin{array}{rcl}{r}_{s1} & = & A+BC,\\ {r}_{s2} & = & A+B(C+D),\end{array}$$

and

8 $$\begin{array}{rcl}{\theta }_{s1} & = & C/f-A/Dccd,\\ {\theta }_{s2} & = & {\theta }_{s1}+D/f,\end{array}$$

where A,B,C, and D are defined as follows

9 $$\begin{array}{rcl}A & = & Dccd({\theta }_{in}-({r}_{in}+p{\theta }_{in})/f),\\ B & = & (Dccd/f-1),\\ C & = & z{\theta }_{in}-({r}_{in}+p{\theta }_{in})(z/f-1)\\  &  & +\,z({\theta }_{in}-({r}_{in}+p{\theta }_{in})/f),\\ D & = & 2\frac{{n}_{1}}{{n}_{2}}t({\theta }_{in}-({r}_{in}+p{\theta }_{in})/f).\end{array}$$

Here, $p$ is the distance between the light source and the interferometer lens L. We used plane wave for incident light; therefore, the incident light angle is zero and the ray equations do not depend on $p$. To form the right interference pattern, ${r}_{s1}$ and ${r}_{s2}$ must be equal:

10 $${r}_{s}\doteq {r}_{s1}={r}_{s2},\,\Rightarrow \,B=0,\,\Rightarrow \,Dccd=f.$$

So, by placing CCD on back focal length of lens L, the compliance condition will be established for all arbitrary incident lights.

### Calculation of the phase differences

The classical optical phase equation $\delta \phi =k.\Delta$ is used to determine the phase difference. Here, $k$ and Δ represent the wavenumber and the optical path length, respectively. According to Fig. 1, Δ can be calculated as the sum of two parts: the optical path length of the specimen Δ_1_, and that of the interference lens Δ_2_:

11 $$\begin{array}{rcl}{\Delta }_{1} & = & \frac{2{n}_{2}t}{\cos ({\theta }_{t})}\\ {\Delta }_{2} & = & 2{n}_{2}t\frac{{\sin }^{2}({\theta }_{t})}{\cos ({\theta }_{t})}\\ \delta \phi ({r}_{s}) & = & k.({\Delta }_{1}({r}_{s})-{\Delta }_{2}({r}_{s}))\\ \delta \phi ({r}_{s}) & = & 2{n}_{2}kt\,\cos ({\theta }_{t})\end{array}$$

## Methodology of thickness measurement

**Figure 2:**
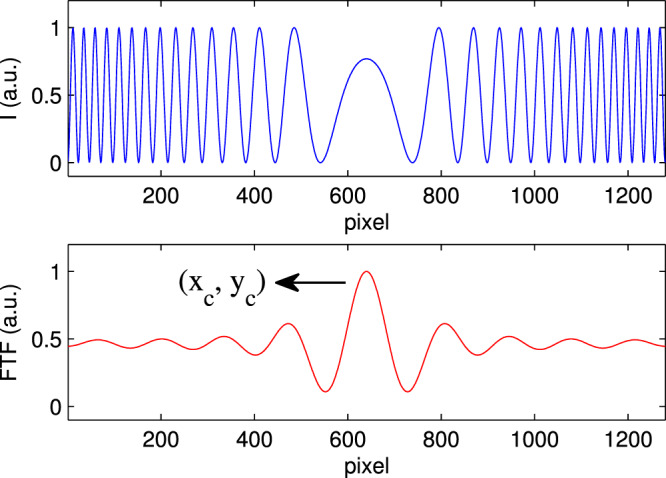
(Up) The cross section, and (Down) the Fourier filtered interference pattern as a function of the position (in pixels), with the determined center point of the fringes (${x}_{c}$, ${y}_{c}$).

Thickness $t$ of transparent plates was measured using Polar Fringe-Tracking (PFT) method. First, by using two-dimensional Fourier transform and bandpass filtering method, the center position of circular fringes (${x}_{c}$, ${y}_{c}$) was extracted. The Fourier filtering of the interference pattern gives a Bessel shape function, so the center of the fringes is equivalent to the position of the intensity peak. Then, by using the interference condition for fringes, the thickness $t$ was calculated. Fig. 2 shows the cross section and the Fourier filtered interference pattern.

### PFT method

In PFT method the position probability density function (PPDF) ${W}_{q}(r)$ of bright/dark fringes ($q=b\,{\rm{or}}\,d$) can be calculated using the polar scanning technique. In this method, the interference pattern is converted to two bright and dark fringe images (${I}_{b}$ and ${I}_{d}$ respectively). Using the quantity ${I}_{thr}=({I}_{b}+{I}_{d})/2$ which indicates intensity threshold, ${I}_{b}$ and ${I}_{d}$ can be written as:

12 $$\begin{array}{l}\{\begin{array}{ll}{I}_{b}=1, & {\rm{if}}\,I\ge {I}_{thr}\\ 0, & {\rm{otherwise}},\end{array}\\ {I}_{d}=1-{I}_{b}.\end{array}$$

By using Eq. 12 in the range of $0\le r\le {r}_{max}$ and $0\le \theta \le 2\pi$, the PPDF can be calculated as (*r*_*max*_, maximum radius):

13 $${W}_{q}(r)=\mathop{\sum }\limits_{\theta =0}^{2\pi }\,{I}_{q}(r,\theta ),\,(q=b\,{\rm{or}}\,d)$$

**Figure 3:**
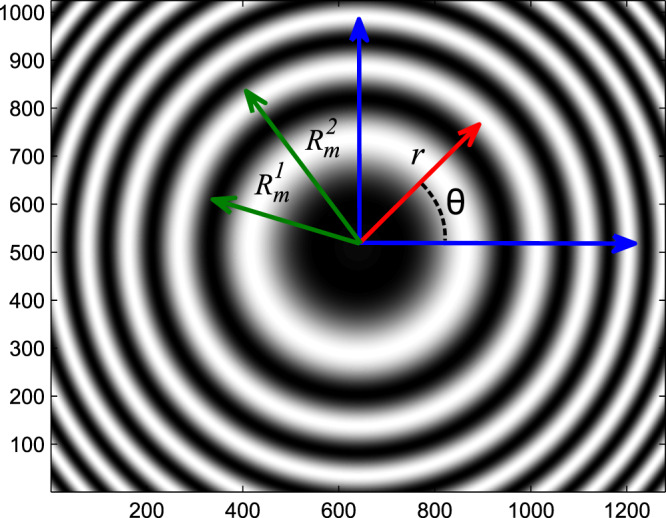
The interference pattern of glass plate ($t=1$ mm), and polar fringe-tracking PFT coordinate.

As shown in Fig. 3 the value of ${W}_{b}$ in range of ${R}_{m}^{1}\le r\le {R}_{m}^{2}$ for one bright fringe (*m*th ring) is equal to the sum of the total number of nonzero points.

**Figure 4:**
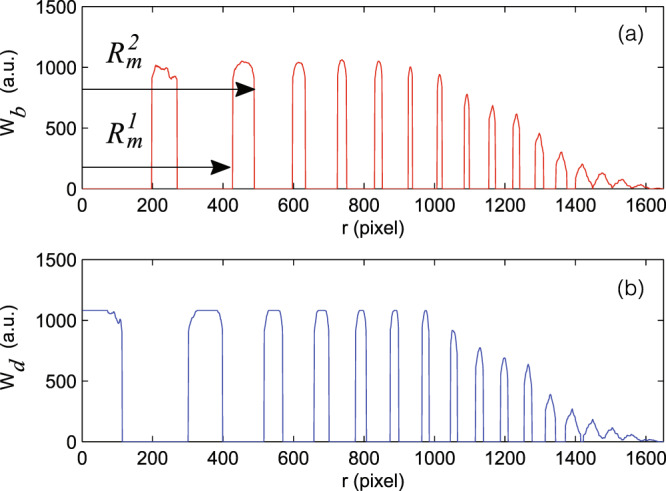
The variation of position probability density function (${W}_{q}(r)$) in terms of distance from the center. (**a**) Dark fringes, (**b**) light fringes.

In Fig. 4, ${W}_{q}(r)$ is shown as a function of $r$. Here, ${r}_{max}=1650$ pixels and $r=0$ is the center of the fringes. To calculate the radius of the interference ring ${R}_{q}(m)$, (*m*th ring), we used the weighted average equation as:

14 $${R}_{q}(m)=\frac{{\sum }_{r={R}_{m}^{1}}^{{R}_{m}^{2}}r.{W}_{q}(r)}{{\sum }_{r={R}_{m}^{1}}^{{R}_{m}^{2}}{W}_{q}(r)}.$$

In this equation, ${R}_{m}^{1}$ and ${R}_{m}^{2}$ represent zero points of ${W}_{q}(r)$ function (Figs. 3 and 4).

### Thickness measurement of a solid plate

The interference condition equations for bright and dark fringes were used to calculate the thickness of a transparent plate (a microscope glass slide).

Using Eq. 11 and interference condition equations related to bright and dark fringe positions (${R}_{q}(m)$), the following equation is obtained:

15 $$\delta \phi ({r}_{s},t)=2{n}_{2}kt\sqrt{\frac{1+\left[1-{\left(\frac{{n}_{1}}{{n}_{2}}\right)}^{2}\right]{\left(\frac{{r}_{s}}{f}\right)}^{2}}{1+{\left(\frac{{r}_{s}}{f}\right)}^{2}}}$$

16 $$\Delta \phi ({r}_{s}^{1},{r}_{s}^{2},t)\doteq \delta \phi ({r}_{s}^{1},t)-\delta \phi ({r}_{s}^{2},t)=2\pi \Delta m.$$

Here, ${r}_{s}^{1}={R}_{q}(m)$ and ${r}_{s}^{2}={R}_{q}(m+\Delta m)$, represent *m*th and ($m+\Delta m$)th fringe positions, respectively. Solving Eq. 16 in terms of $t$, for different separation numbers Δ*m*, the plate thickness is extracted. To increase the accuracy of the measurement, by using linear interpolation method, the distance between two consecutive fringes (${R}_{q}(m)$ and ${R}_{q}(m+1)$) is divided into ${N}_{0}$ parts, so the Eq. 16 becomes ($\Delta m{\prime} =1,2,3,\ldots ,{N}_{0}\Delta {m}_{max}$)

17 $$\Delta \phi ({r}_{s}^{1},{r}_{s}^{2},t)=\frac{2\pi }{{N}_{0}}\Delta m{\prime} .$$

**Figure 5:**
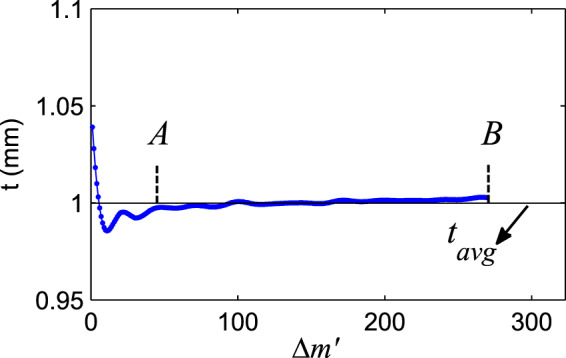
Experimental thickness variation in terms of sub-index Δ*m*′.

Fig. 5 shows the variation of the measured layer thickness $t$ vs. $\Delta m{\prime}$ with $\Delta {m}_{max}=27$ and ${N}_{0}=10$. As shown in Fig. 5, the variation of $t$ has the fluctuation behaviour around the mean value ${t}_{avg}$ for small separation sub index $\Delta m{\prime}$ ($\Delta m{\prime}  < 50$).

**Figure 6:**
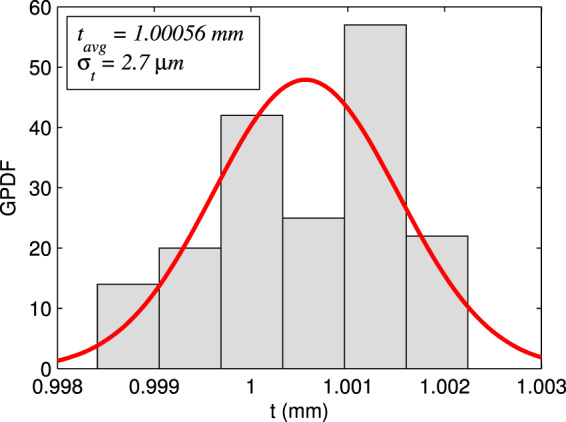
The Gaussian probability density function (GPDF) of thickness $t$. The mean value ${t}_{avg}=1.00056$ mm and standard deviation ${\sigma }_{t}=2.7$ *μ*m are determined.

We used Gaussian probability density function (GPDF) for thickness $t$, for determination of the mean value (${t}_{avg}$) and standard deviation (${\sigma }_{t}$) of measurements in the linear region (AB in Fig. 5). In Fig. 6 the GPDF of a sample with $t=1$ mm and ${n}_{2}=1.5214$ is shown. In this measurement the average thickness is ${t}_{avg}=1.00056$ mm and the standard deviation is ${\sigma }_{t}=2.7$ *μ*m.

We used standard glasses, to test our method. The thicknesses of standard glasses have been reported by their manufacturer (Edmund Glasses). In Table 1, the thickness of the standard glasses measured by PFT method is compared with the value reported by the factory. It shows that the measured thicknesses are in a very good agreement with the reported amounts. The advantage of this method is the thickness measurement in a wide range of 0.01 to 3 mm, with an accuracy of 100 nm. Until now, there has been no method for thickness measurement in this rage and with this accuracy. This method can be used for measuring the thickness of transparent solids and liquids, which is of great importance in industrial applications and research problems.

**Table 1:** The reported and measured values for the thickness of standard glass plates.

Glass Type	Refractive Index	Reported Thickness	Measured Thickness	error (%)
Edmund 47681	1.434	1.5 mm	1.4988 mm	0.008
Edmund 02105	1.434	1.5 mm	1.4823 mm	1.2
Soda Lime	1.5214	1 mm	1.0548 mm	5.5

### Thickness measurement of a film on a substrate

To measure the film thickness on a transparent substrate, we can use PSRI. Combining the phase difference Eq. 15 and the thickness Eq. 17, the following equation is obtained for the double layer system

18 $$\delta \phi ({r}_{s},{t}_{1},{t}_{2})=\delta \phi ({r}_{s},{t}_{1})+\delta \phi ({r}_{s},{t}_{2}).$$

In reconstruction of Eq. 18, we have ignored the interference caused by the boundary between the two layers, considering the difference of two refractive index values is very small compared to the value of each one. So, the intensity of reflected light from the boundary is weak and does not affect the final interference pattern.

## Experimental results of liquid film thickness

### Spin coating

**Figure 7:**
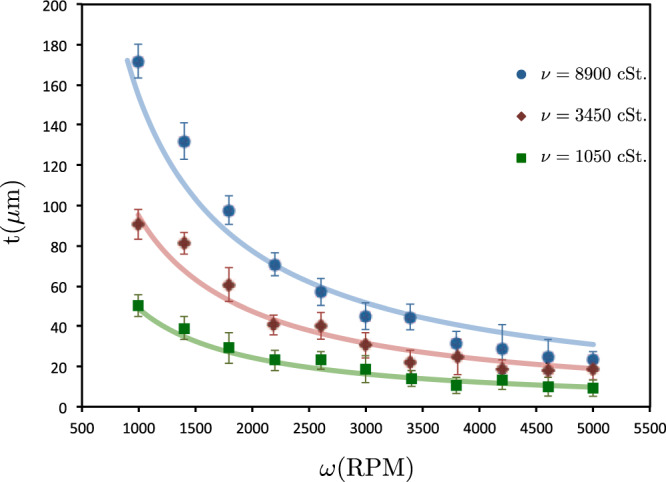
The silicone oil thin film thickness after 30 s in terms of spin coating velocity for different viscosities (squares: 1050 cSt, diamonds: 3410 cSt and circles: 8900 cSt). The lines show the fitting curves proportional to ${\omega }^{-1}$.

In Fig. 7 the measured thickness of silicone oil thin films prepared with spin coating method is shown. We used a glass substrate with the refractive index of ${n}_{g}=1.5214$ and the thickness of ${t}_{g}=1$ mm; with different spin coating velocities $V$ in the range of 1000 to 5000 RPM (rounds per minute) with steps of 400 RPM.

In this experiment, three kinds of silicone oil with different viscosities ($\eta =1050$, 3450, and 8900 cSt.) and refractive index of ${n}_{l}=1.408$ have been used. The results show the power law thickness variation in terms of spin coating velocity, which is in good agreement with similar reports^15,16^.

According to Fig. 7, the average standard deviation in the range of 7 *μ*m to 25 *μ*m. Due to the repetitive data collection for each sample (ten times in each experiment) and stochastic behavior of spin coating technique, the estimated average error is large compared to the error of each experiment which is less than 3 *μ*m.

### Dip coating

Although there are accurate experimental measurements of the liquid film thickness in LLD regime ($Ca < 0.001$) for dip coating^25^, there is no accurate experiment for $Ca > 0.001$, where the effect of gravity becomes important. Maleki *et al*.^25^ used the method of weighing for larger $Ca$, which is not accurate due to the drainage and edge effects. Here, we report reliable experimental data for $Ca$ up to 0.3, for the first time.

**Figure 8:**
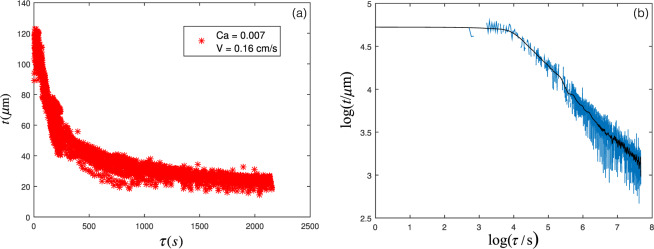
The measured thickness of liquid film as a function of time for *V* = 0.16 cm/s and *Ca* = 0.007: (**a**) normal scale, and (**b**) logarithmic scale, with moving average (black curve).

The PFT method is a good method to measure the thickness of liquid thin films in a non-contact, non-destructive and immediate way. Microscope slide glasses (QC LAB 7102) of dimensions 76 × 25 × 0.1 mm were used as glass substrate. The glass substrates were cleaned in commercial detergent, rinsed well in distilled water and then dried in open air before use. The glass substrate was kept by a holder in front of a He-Ne laser light beam that passed through the PFT setup. The glass substrate was fixed, and the silicon oil container moved up and down with a servo motor and a mechanical setup. The interference patterns were recorded by a CCD camera (Compact USB 2.0 CMOS Camera, THORLABS) with the rate of 25 frames per second. The film was extracted into frames and then analyzed by PSRI method. Fig. 8(a) shows the results for the thickness of the liquid thin film as a function of time. The advantage of the PFT method is being able to measure the thickness in each frame and without any time measurement, and the time resolution is given by the frame rate of the camera, without limitation. Its problem is the dispersion of the data. This can be solved by moving average procedure on the data, which gives a more smooth plot for the thickness as can be seen in Fig. 8(b). The viscosity of the silicon oil was 100 cSt and the glass substrate was pulled out of a silicon oil bath with the velocity of 1.6 mm/s, thus the capillary number was about 0.007. The thinning due to the gravitational entrainment can be observed. It can be seen in Fig. 8(b). The figure also shows that first we have a film with a constant thickness, and then starts to thin due to gravity, as we expect from Eq. 5.

**Figure 9:**
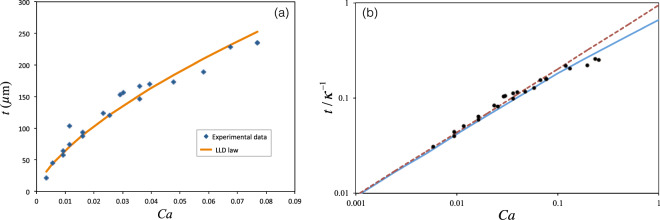
The initial thickness of the film as a function of *Ca*. (**a**) For $Ca < 0.1$,the symbols: experimental results, and the curve: LLD law. (**b**) The rescaled thickness as a function of *Ca* in log-log scale; symbols: experiments, dashed line: LLD law, solid line: Jin - Acrivos theory.

The initial thickness of the film, extracted from the constant part of the thickness curve vs. time, was plotted as a function of $Ca$. Based on theories, we expect that in low $Ca$, the thickness should follow the LLD law. Fig. 9(a) shows the experimental results for $Ca < 0.1$ and the theoretical LLD curve. There is a good agreement between the experiments and the theory.

For higher capillary numbers, we should see a deviation from the LLD law, because of the gravitational effect. As shown in Fig. 9(b), for $Ca\sim 0.1$ the deviation is clearly observable, and the behavior is consistent with Jin and Arivos calculations^22^.

**Figure 10:**
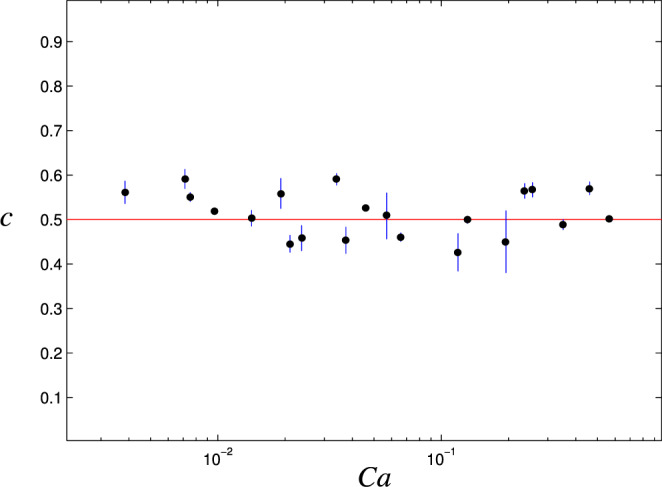
The thinning power *c* as a function of *Ca* obtained from experiments.

The thinning due to gravity was observed in all the experiments. We fitted a power-law curve $t\propto {\tau }^{-c}$ to the decreasing part of the thickness vs. time plot (see Fig. 8(b)) and measured the thinning power, which should be 0.5 based on the theory (Eq. 5). As observed in Fig. 10, the measured thinning power is in fact close to 0.5 for all the experiments in all $Ca$.

## Conclusions

The thickness of transparent layers and spin coated liquids has been studied as a function of optical parameters. We determined the accurate optical parameters for thickness measurement by using the paraxial approximation and ray tracing calculations. The PFT method is introduced to reduce the interference noises and measurement errors. The linear interpolation technique has been used to increase the total number of mid points. Therefore, by using the Gaussian probability density function (GPDF) the mean value and standard deviation of experimental data are extracted. The Paraxial Self-Reference Interferometer (PSRI) introduced in this paper is a useful tool for thickness measurements in many well-known optical systems. We used the method for two kinds of liquid film experiments and showed that the thickness of the films can be measured with a high accuracy in a wide range of thicknesses. Its other advantage is the high time resolution which can be suitable for dynamic systems with thicknesses varying with time.
